# A Case Report of Acute Lymphoblastic Leukemia Presenting as Bilateral Knee Pain in a Healthy Runner

**DOI:** 10.5811/cpcem.47307

**Published:** 2026-01-05

**Authors:** Michael Rosselli, Alejandro Sanoja, Matthew Apicella

**Affiliations:** * Mount Sinai Medical Center, Department of Emergency Medicine, Miami Beach, Florida; † University of Florida, Department of Emergency Medicine, Gainesville, Florida

**Keywords:** acute lymphoblastic leukemia, sports medicine, emergency medicine, case report

## Abstract

**Introduction:**

Acute lymphoblastic leukemia (ALL) is typically a childhood disease but may present in older patients in rare occurrences. Due to its significant morbidity and mortality, early diagnosis is crucial. The symptoms of ALL may be non-specific, making the initial diagnosis difficult leading to delayed treatment.

**Case Report:**

We present the case of a 34-year-old, healthy male runner presenting to the emergency department with a common complaint of bilateral knee pain, who was ultimately diagnosed with ALL with signs of tumor lysis syndrome leading to premature death.

**Conclusion:**

We discuss the presenting symptoms of acute lymphoblastic leukemia, which may include joint or knee pain as well as leukemic arthritis. We further discuss the importance of clinicians maintaining a high level of suspicion for the “bounce-back” patient and avoiding taking cognitive shortcuts.

## INTRODUCTION

Acute lymphoblastic leukemia (ALL) accounts for 12% of all leukemia cases worldwide.^1^ It is normally a disease of childhood with typical presentation in children < 6 years of age, although a second peak of incidence typically occurs at ages > 60.^1^ The presenting symptoms of ALL are often vague but can include joint and bone pain.^1^,^2^ This case is unique in that the disease presented in an otherwise healthy, 34-year-old active individual.

## CASE REPORT

We present the case of a 34-year-old, previously healthy male runner who presented to the emergency department (ED) for intermittent bilateral posterior knee pain for one month, worse in the morning after he awoke. The patient had been evaluated twice in the ED since the onset of his symptoms and discharged home each time with orthopedic follow-up, which he did not follow through with due to time constraints secondary to his job. Radiographs performed of the bilateral knees on his previous visit were remarkable only for mild bilateral joint effusions, without fracture or evidence of arthritic changes. He also. Underwent an unremarkable duplex ultrasound of the bilateral lower extremities.

Considering he was an avid runner and worked in construction, the treating physicians on prior visits concluded that his symptoms were likely musculoskeletal in etiology. The patient denied a history of fever/chills, easy bleeding or bruising, night sweats, and weight loss. Physical exam findings on our evaluation were negative for any strength deficits, joint laxity, restricted range of motion, swelling, warmth, or skin changes. He stated that he took ibuprofen, which normally relieved his symptoms. When the pain persisted he returned to the ED a third time.

Bloodwork was drawn, and radiographs repeated on this visit were again found to be unremarkable. Blood analyses revealed a white blood cell count of 395,000 microliters (μL) with 33% blasts (reference range: 4.80–10.80 10*3/μL), hemoglobin of 3.9 grams per deciliter (g/dL) (12.0–16.0 g/dL), and a platelet count of 38,000 μL (reference range: 150–450 10*3/μL). The patient was also noted to have an acute kidney injury with a creatinine of 1.35 milligrams (mg)/dL (0.55–1.02 mg/dL), potassium of 8.7 millimoles per liter (mmol/L) (3.5–5.1 mmol/L), phosphorus of 4.5 mg/dL (2.5–4.5 mg/dL), calcium of 8.0 mg/dL (8.5–10.1 mg/dL), and uric acid level of 11.7 mg/dL (reference range: 3.4–7.0 mg/dL). The electrocardiogram showed normal sinus rhythm with no significant T wave changes.

He was transfused with two units of packed red blood cells and treated for his hyperkalemia with one gram of calcium gluconate, 10 units of regular insulin with 1 ampule of D50, 50 milliequivalents of sodium bicarbonate, and 2.5 mg of albuterol nebulizer. He was also given 4.5 mg of rasburicase and two liters of normal saline for concern of tumor lysis syndrome. All medications and fluid boluses were given once. Oncology and nephrology were consulted from the ED. Both services agreed with current management and recommended admission to the intensive care unit (ICU) for continued management and close observation. The patient was admitted to the hospital and ultimately diagnosed with acute lymphoblastic leukemia based on cytology and positive Philadelphia chromosome.

Six days into his admission, the patient suffered a large, right-sided spontaneous intraparenchymal hemorrhage in the setting of severe thrombocytopenia; platelets were 7,000 μL. He required emergent craniotomy and intubation. His course was further complicated by aspiration pneumonia. Bronchioalveolar lavage and computed tomography of the chest led to concern for cavitary aspergilloma, requiring intravenous antifungals, broad-spectrum antibiotics, and pressor support for septic shock. The patient ultimately required tracheostomy, percutaneous gastric tube placement, and inferior vena cava filter as he was bedridden and unable to take anticoagulants. After his prolonged ICU course, and prior to his discharge, he was started on methotrexate, nilotinib, and ponatinib.

Per family, the patient followed up with oncology after discharge and failed chemotherapy at an outside hospital. We were unable to obtain records of these failed treatments. The patient continued to have seizures after his discharge and was put on daily levetiracetam and lacosamide. He was unable to work in construction and became dependent on family for activities of daily living. He ultimately died two years after his diagnosis was made. He is survived by his parents who took the time to speak with us in detail about their son after he had died.

## DISCUSSION

Acute lymphoblastic leukemia typically has a bimodal distribution, presenting in children < 6 years of age and adults > 60.^3^ The incidence of ALL in the United States is estimated at 1.6 per 100,000 population, with > 1,400 deaths annually.^3^ The disease presentation is more devastating in adults.^3^ It involves the abnormal proliferation and differentiation of lymphoid cells, and clinical manifestations typically presents when these cells accumulate in the bone marrow, peripheral blood, and extramedullary sites.^3^

*CPC-EM Capsule*

What do we already know about this clinical entity?

*Acute lymphocytic leukemia (ALL) is a cancer of lymphoid cells, most commonly affecting children < 6 and adults > 60, with the most common complaint being fatigue.*

What makes this presentation of disease reportable?

*A 34-year-old male, status post multiple visits to the emergency department with the complaint of bilateral knee pain without fatigue, fever, bleeding or bruising, was diagnosed with ALL.*

What is the major learning point?

*Cognitive biases may contribute to delayed diagnosis when otherwise healthy “bounce-back” patients return to the emergency department with the same complaint.*

How might this improve emergency medicine practice?

*This case highlights the need to remain vigilant for cognitive biases and to generate a broad differential for all patients.*

The presentation of ALL is often non-specific and includes classic “B symptoms”—fever, weight loss, and night sweats—along with symptoms of easy bleeding, bruising, lymphadenopathy, joint pain, and bone pain.^3^ Although our patient did not have classic B symptoms he did experience progressively worsening joint pain. Diagnosis is established by the presence of ≥ 20% lymphoblasts in the bone marrow or peripheral blood. Further testing typically includes bone marrow biopsy, cytology, and genetic testing.^3^ Coagulation studies, renal function studies, and electrolytes should concurrently be investigated as the patient should be evaluated for tumor lysis syndrome, as was seen in our patient, which is an oncologic emergency associated with underlying malignancies with a high degree of mortality. It should be treated immediately.^4^

It should be noted that although joint aspiration was not done on our patient, it could be considered in cases of leukemia-associated joint pain to assess for leukemic arthritis.^5^ In our patient’s prior ED visit, he presented with mild effusions seen on knee radiographs. Joint aspiration could have been done, as immunohistology studies of synovial fluid may aid in diagnosis as would synovial biopsy.^5^ Prior cases of leukemic arthritis have shown blast cells within synovial fluid.^5^,^6^ Leukemic arthritis is a well-recognized complication in children but may also be the presenting symptom in adults with a frequency of 14% and 4%, respectively; it is more commonly associated with larger joints such as knees and shoulders.^5^,^7^

It is also important to consider cognitive biases associated with delayed diagnosis in the otherwise healthy “bounce-back” patient. Such biases include anchoring bias, halo effect, perception bias, and premature closure (Table).^8^ Anchoring bias is particularly important to consider in the ED. It has been shown that emergency physicians at all levels of training are susceptible to anchoring bias.^8^,^9^ Studies cite increased pressure to see patients rapidly, triage note documentation, and the high-pressure nature of the job as factors that may influence emergency physician decision-making.^8^–^10^ Zandbergen et al showed that anchoring bias in resident physicians may be limited by increased time spent deliberating a differential diagnosis. Our case exemplifies how cognitive bias may impact patient care and the discriminating features of ALL (joint pain in an otherwise healthy young male).

## CONCLUSION

This atypical case presentation highlights the importance of having a high degree of suspicion for a more severe disease process in the bounce-back patient. The outcome of this case highlights the importance of doing so even in a seemingly benign complaint of bilateral knee pain in an otherwise healthy runner. It also underscores how delayed diagnosis may lead to poor prognosis and tragic outcomes, especially when we fail to consider cognitive biases in the medical decision-making process.

## Figures and Tables

**Table:** Types of cognitive bias,

Cognitive Bias	Definition and example
Anchoring bias	Too much weight is given to the early and most prominent feature of a patient’s history, physical, or test results. This causes diagnostic momentum in favor of a certain diagnosis. For example, a triage note states that a patient with heart failure has shortness of breath with leg swelling. The emergency practitioner fails to consider evaluating the patient for pulmonary embolism in history-taking and instead continues to do a work-up for heart failure.
Premature closure	The clinician stops at a plausible diagnosis despite an incomplete work-up. An example would be a clinician who discharges a patient home with the diagnosis of a urinary tract infection based on the urinalysis alone, without considering acute appendicitis in that patient with right lower abdominal pain with urinary symptoms.
Halo effect	Tendency to let the first impression of a person influence one’s thoughts about the individual overall. Example: A clinician in the office downplays a patient’s abdominal pain, stating “this patient tends to overreact with everything.”
Perception bias	Tendency to stereotype and assume beliefs about a group of people and imposing those beliefs on an individual who belongs to that group. Example: A physician states, “Soccer players tend to be a histrionic group,” when discussing a patient.
Ascertainment bias	In clinical practice, this refers to a clinician’s tendency to base their work-up on prior clinical experiences. Example: A chronic undomiciled patient with multiple presentations of alcohol use disorder arrives to the emergency department unconscious, and the clinician assumes it is acute alcohol intoxication due to prior experience and performs a limited neurologic exam only to find that the patient ultimately had a massive intracranial hemorrhage.
